# An Empirical Study on Emergency of Distant Tertiary Education in the Southern Region of Bangladesh during COVID-19: Policy Implication

**DOI:** 10.3390/ijerph20054372

**Published:** 2023-02-28

**Authors:** Md. Abu Issa Gazi, Abdullah Al Masud, Farid Ahammad Sobhani, Bablu Kumar Dhar, Mohammad Sabbir Hossain, Abu Ishaque Hossain

**Affiliations:** 1 School of Management, Jiujiang University, Jiujiang 332005, China; 2 Department of Management Studies, University of Barishal, Barishal 8254, Bangladesh; 3 School of Business and Economics, United International University, Dhaka 1212, Bangladesh; 4 Mahidol University International College, Mahidol University, Nakhon Pathom 73170, Thailand; 5 Department of Finance and Banking, Patuakhali Science and Technology University, Patuakhali 8602, Bangladesh; 6 Department of Business Administration, The International University of Scholars, Dhaka 1213, Bangladesh

**Keywords:** COVID-19, higher education, students learning, experience, teaching

## Abstract

Many fields have been affected by COVID-19, including education. The pandemic has prompted a change in education due to the requirement for social distancing. Campuses are now closed in many educational institutions across the globe, and teaching and learning are now conducted online. Internationalization has significantly slowed down. A mixed-method study was designed for this research, with the goal of ascertaining the impact of COVID-19 on Bangladeshi students enrolled in higher education during and after the pandemic. A questionnaire with 19 questions on a Google form was used to collect quantitative data using a 4-point Likert scale and was conducted on 100 students from different universities in the southern part of Bangladesh, such as Barisal University, Patuakhali Science and Technology University, and Bangabandhu Sheikh Mujibur Rahman Science and Technology University. For collecting qualitative data, six quasi-interviews were conducted. A statistical package for Social Science (SPSS) was used to analyze both quantitative and qualitative data. The quantitative results demonstrated that during the COVID-19 pandemic, pupils continuously received teaching and learning. The current study’s findings revealed a significant positive correlation between the COVID-19 pandemic and teaching, learning, and student achievement and a significant negative correlation between the COVID-19 pandemic and student goals. The study also revealed that the COVID-19 pandemic had a detrimental effect on students enrolled in higher education programs at the universities. The qualitative judgment showed that students faced many problems when joining classes, such as poor Internet connection and insufficient network and technological facilities, etc. Some students live in rural areas and have slow Internet speeds, which sometimes prevented them from joining class. The findings of the study can help policy makers in higher education to review and adopt a new policy in higher education in Bangladesh. It can also help education instructors in universities to develop a proper study plan for their students.

## 1. Introduction

The worldwide pandemic originated in the Chinese town of Wuhan and spread to the whole world. SARS is a newly developed virus that belongs to the coronavirus family [1]. Many believe that the coronavirus is an infectious disease caused by animals and humans [2,3]. The infection spread first in Wuhan in December 2019 and January 2020 and then spread around the world after February, resulting in the continued 2019–2020 COVID-19 pandemic [2,4]. The World Health Organization declared the COVID-19 pandemic on 11 March 2020 [2]. A new variant of COVID-19 called Omicron has been identified [2,5,6,7,8]. The COVID-19 pandemic was first detected in Bangladesh on 8 March 2020, when a couple of men and a woman, aged 20 and 35, were diagnosed with the disease [9]. The country’s first death from the virus occurred on 18 March 2020, when a 70-year-old man died [10]. The pandemic has affected every aspect of human life, and its true impact is still unknown, as the spread of the disease, its severity, death rate, policy responses, and individual behavior are all uncertain [10,11,12,13]. It affects every country regardless of its race, nationality, and economic status [10]. COVID-19 has wreaked havoc on poor nations such as Bangladesh, with an estimated 40 million students missing school until the virus is brought under control [12,13,14]. Since March, the spread of COVID-19 has increased daily. To prevent its spread, the government of Bangladesh has adopted social distancing, lockdown, and community containment measures from March 2020 onward [15,16,17,18]. The most important pandemic precaution, referred to as “social distancing” or “physical separation”, was implemented to restrict social contact and prevent the type of communal infection that can quickly spread in extensive social networks such as university premises [9]. Since 17 March 2020, the Bangladesh government has closed all educational institutions and imposed a ban on important meetings as a first-line emergency response to prevent the mass dissemination of COVID-19 [9,19]. Due to public concern, the closure period was extended several times, and it presently runs until 15 June 2020 [19]. The COVID-19 outbreak has had a negative impact on education globally. Around 1.6 billion students in 200 nations are impacted by COVID-19 pandemic-related school cancellations [2]. According to Jaime Saavedra, Global Director for Education, it is the “biggest simultaneous shock to all education systems in our lifetimes” (World Bank, 2020). News sources report that there are about 30 million students in various institutions in Bangladesh as well as around a million teachers and education personnel [4,19].

University students in Bangladesh have suffered immensely due to the loss of their academic and social lives, which has had a negative influence on their mental health [4,20]. To proceed with the customary pace of instruction and limit the shortage, the nation has turned to online classes, but it is questionable whether online classes are an identical substitute to the genuine actual classroom environment [17,21]. This is because, keeping other tricky issues aside, students find it more challenging to appreciate and grasp the items in web-based contexts than in a genuine classroom [17,22]. The University Grants Commission (UGC) held a meeting on 25 June 2020 to meet with the top administrators from public colleges to discuss the transition to online teaching through Zoom and Google Meet to close this gap. The decision focused on merely starting online sessions and deferring exams and lab activities, placing students in a condition of great uncertainty [18,22]. This transition was one of the most difficult for developing countries, as they lacked adequate infrastructure, faced financial constraints, and had defects in educational policies that made it difficult to provide enough facilities for online instruction. This may indicate that there is educational inequality among students, particularly those in university education, in Bangladesh and throughout the world [23]. Moreover, students’ inability to bear the cost of a work area or computer with important technology, especially through state-funded colleges, which are funded largely from monetary imperatives, is viewed as one more significant misfortune for online classes in Bangladesh [24,25]. In fact, in Bangladesh, only 36.7% of families have access to the Internet, and just 5.6% have their own computer facilities [26,27]. Furthermore, students and teachers have faced a variety of challenges, including a lack of hardware, poor Internet access, expensive data packages, and adapting to a new learning environment [20]. Worldwide, 50% of students (826 million) do not have access to a computer, and 43% (706 million) do not have an Internet connection. In addition, nearly 56 million pupils are unable to use portable devices due to the lack of mobile version presence. Several times, students have failed to log into the particular online learning program, indicating poor student involvement in online learning [27,28]. Due to the COVID-19 pandemic, there are significant problems in every aspect of human existence. The nationwide closure has halted workouts, causing harm to the school system and facilities. The disease’s transmission is accelerating, and it has a considerable impact on academic endeavors, particularly in higher education. The study analyzes the impact of this pandemic issue. This is a serious issue that affects not only the students but also the teachers. Further research into the problems related to how this affects university education’s learning systems will help improve our understanding of the actual impact and students’ views as well as strategies that can be employed to combat it and help regulate the effectiveness of professional learning.

As a result, no study has been conducted to determine how COVID-19 will affect degree learning in Bangladesh. Consequently, to the best of our knowledge, we conducted a mixed study to examine the actual impact of COVID-19 on students studying there. There is a disparity in higher education in Bangladesh, and this study seeks to reduce it, and it will likewise support straining leader to create and execute viable schooling COVID-19 pandemic responses. Further study may be needed to achieve better output because this study is limited by sufficient data.

This study aims to examine the impact of the COVID-19 pandemic on student learning in higher education in Bangladesh. The analysis has specific objectives including the impact of the COVID-19 pandemic on the higher education system in Bangladesh, teachers’ and students’ attitudes towards online classes, and the students’ experience with online classes. The other main objective is to measure the actual impact of COVID-19 on university student learning.

## 2. Literature Review

The worldwide, relentless pandemic of COVID-19 has already had devastating impacts on every aspect of civilization, including business, culture, and education. The pandemic has caused and continues to bring about a long-term economic deficit [29]. However, in a country such as Bangladesh, the loss of the educational sector is more crucial than any other deciding factor. It has brought the teaching-learning arena’s regular progress to a screeching halt [30,31]. Governments are powerless to find cures, protectants, and antidotes for viruses that have a profound impact on human life [18]. Throughout most countries, schools, training institutes, and further education facilities have been closed as a result of COVID-19 lockdown and social distancing measures. As educational institutions close, there is a need to take virtual classes and exams [32]. Education and learning are one of the most important needs of society, and this pandemic problem makes it difficult for teachers and students to continue their courses [18]. A fundamental shift is taking place in how educators deliver high-quality education [12,13,33]. It was easy for educational institutions to provide classes occasionally on online platforms based on distance education mode. Because of the pandemic, these platforms were the only way to hold classes with a large number of students [34]. The COVID-19 process has altered the way that education is viewed and understood. Whenever the studies on the pandemic period’s distance education process are examined, it becomes clear that researchers discuss both the advantages and disadvantages of distance learning and education [35,36,37]. The advantages of the distance education were assessed during the pandemic. These advantages included updated lessons plan, efficient tools, the visual melted lessons, ease of access to the resources, cost-effectiveness, and learning at students’ own pace [36]. Technical issues, forgetfulness, lack of interaction, difficulties encountered during the pandemic process, a large number of students, and low motivation were also considered to be detrimental aspects of distance education and learning as conducted during the pandemic [38]. In addition to the drawbacks of distance education, there are instances in which socialization with it is impeded, applied lessons are insufficiently effective, and the necessary assistance cannot be given to students who are unable to learn on their own [39].

The challenges of the global epidemic forced all educational institutions, including universities, to act quickly and shift the educational process to blended or distance learning and flexible learning [38]. Online learning and distance learning are two conceptually opposed theories of learning that are used in contemporary pedagogical education [40]. Online education frequently takes on a forced form and heavily mimics classroom instruction. It has benefits such as making presentations and video materials easier to demonstrate, conducting online tests, etc. [41]. Learning through distance education has a fundamentally different structure and method of communication. In distance learning, teachers may not interact with students in any ways during online broadcasts and will only do so if it is absolutely necessary [42]. A huge array of technological tools is available for distance learning, including audio podcasts, videos, different simulation software, and online tests [43]. To build each student’s unique trajectory, a meticulous tracking of their performance is the significant priority of distance learning [40]. In times of emergency, distance learning is used, and traditional classroom-based curricula are developed along with the subjects and programs [38]. Emergency distance education and learning may be temporarily used in “emergency mode”, but if such an approach becomes a “new reality”, it may be worthwhile to consider making the switch to online education with its regulations, laws, methodology, procedures, and practices. This process requires a great deal of effort and is not straightforward [41].

According to Xie and Rice [44], there are two types of distance learning that are based on how the educational environment is communicated and how information is transferred. Traditional distance learning is the first type; it is an extramural form of learning. E-distance learning is the second type; it is pigeonholed by both asynchronous and synchronous interaction among participants and the educational process’s organizers, and it predominates the profound use of e-learning structures, the Internet, multimedia training tools, and ICT [44]. Yilmaz et al. [40] stated that distance education and learning is a system that is fundamentally new. They list the essential elements of the distance learning arrangement, such as the profound learning platform with the required communication tools, the repository of teaching materials, tracking and evaluation methods, the distance learners themselves, and technical experts such as administrators and programmers.

It is important when studying the phenomenon of distance learning and education to analyze the understanding of the world of open or flexible education and learning that has been employed in many scientific studies [35,38,42]. If distance education is formed to increase admittance to education for those who have not been able to obtain it for a variety of reasons, then the methods of flexible or open education are primarily designed to raise the standard of education [41].Therefore, flexible education, in contrast to distance education, is primarily focused on finding new teaching strategies [42] and modernizing the planning and structuring of the educational process [39], and new technologies are significant only in the context of their innovative application in the educational system [38]. According to Ferrer et al. [45], flexible learning denotes a collection of educational philosophies and systems that concentrate on giving students more choice, relaxation, and personalization to suit their requirements. Flexible learning particularly gives students choices for how, where, and when learning takes place. It is also known as personalized learning which on occasionally has hastened distance education.

E-learning methods have a prominent role to play in accelerating distance education. It can be said that e-learning has emerged as a boon for distance education during the pandemic period [46]. Narvekar [39] stated that to ensure the efficiency of knowledge acquisition, e-learning primarily makes use of Web- and Internet-based technologies and is founded on guiding principles of studying done over a network, and a computer and standard Internet technologies are used to deliver educational content to the end user. Meletiou-Mavrotheris et al. [43] discovered that students’ familiarity with and efficacy with e-learning tools do not necessarily translate into the student becoming a digital learner with the necessary digital skills, such self-regulation, to benefit immensely from online learning. Web learning and online learning are frequently used as synonyms for e-learning. As a result, the phrase refers to the extensive use of Internet connection, computers, and multimedia and remote communication systems in education, especially in distance education [45].

The extent of e-learning has led to emergence of a new kind of learning—blended learning—to enhance distance education [46]. One of the most widely used technologies today is blended learning, which enables learners to combine the advantages of traditional classroom instruction with the flexibility and convenience of a distance learning course [47]. According to Misirli and Ergulec [48], blended learning is a teaching strategy that combines the effectiveness and interpersonal interactions possibilities of the conventional face-to-face classroom with the learning opportunities provided by the online mode of delivery.

In the future, it is anticipated that e-learning, blended learning, open and flexible learning, and distance learning will permeate even more deeply into students’ personal and academic life [47]. Assessment and evaluation of higher education institutions’ and educational systems’ responses to the pandemic are necessary so that wise decisions can be made regarding subsequent actions [43].

To keep up with the routine supervision of learning and to address the shortfall, Bangladesh has moved to e-learning. However, it is still unclear if online programs can completely replace traditional classroom settings. Apart from other concerns, understanding and grasping the material of online lectures is far more difficult for students than it is in a traditional classroom [30,49]. Both students and teachers face a new experience when changing normal face-to-face instruction to digital learning; they have few or no other options [33]. A number of technological challenges are also faced by educators and students. The pandemic revealed significant gaps and variations in educators’ levels of readiness to use technology, teach remotely, as well as to create and deliver distance learning environments [38,39]. The majority of university students come from rural areas. As the government declared a public holiday, the students went back to their homes. During the COVID-19 period, it was necessary to participate in online classes from remote regions [1,50]. In terms of the urban area, the students from rural areas face many problems in online-based classes. In rural areas, broadband connections are not available, and if they are available in some areas, they are very costly. An investigation that included fifty university students determined the effect of COVID-19 on Bangladeshi higher education, revealing realities such as “a loss in motivation and study hours, as well as many physical, mental, and economic concerns related to academic studies. Electronic devices are scarce, internet access is limited, internet costs are high, internet speeds are slow, and online platforms are difficult to use” [4,20]. Another study conducted in Bangladesh revealed similar learner realities such as students’ incapacity to purchase broadband Internet, higher internet data prices, and so on [3]. As the economy of Bangladesh is not developed, the socio-economic condition of the students is deplorable, and most of the students live in villages where they are deprived of technological facilities [14]. Due to the sudden change in education due to COVID-19, students face difficulties, especially not being able to afford technology-supportive devices, lack of Internet coverage, not being able to afford high-priced Internet, and not having satisfactory Internet speed [35]. The main obstacles and concerns were “students’ lack of access to proper gadgets, inconsistent internet connectivity, and high internet costs” and “teachers’ and students’ lack of training and technological skills” [10]. A survey of 416 students from several universities in Bangladesh showed that most students have a favorable attitude towards online education [51]. In a survey of English study students in Indonesia, it was discovered that students’ perceptions of online learning are good, as they believe it is very beneficial during the time of COVID-19. Student perceptions of e-learning were also discovered to “help improve the interactivity between student and teacher”; that it is a “faster way to get feedback”; that “using mobile devices, students can get a response from their teachers at any time, whether it is office hour or not”; and that “the use of social media helps to strengthen the communication” [52,53]. The advantages of remote learning may be seen by those who are unable to attend a traditional face-to-face university because of their personal or financial circumstances [54]. As a result of COVID-19’s interruptions to the learning trajectory, institutions, teachers, and students will continue to seek flexible solutions. Those who are part of the open schools system (e.g., India’s National Institute of Open Schooling and the New Zealand Correspondence School) and open universities (e.g., The U.K. Open University; Athabasca University, Canada)—most of whom have continued to operate through the COVID-19 outbreak—can sometimes help students regain momentum by offering a wide range of courses and flexibility in scheduling and location. The COVID-19 pandemic’s infectious sickness has had an impact on many facets of human existence, including commerce, instruction, health, economics, recreation, transportation, and social relations as well as politics and entertainment [55]. Notably, the environment has been stressful for everyone, and the virus spread has created issues in the educational system [22]. The Bangladeshi government implemented certain efforts to stop the spread of COVID-19. They forbade people from going from one city to another, shut down all educational institutions, and secured the cities [55]. The Ministry of Higher Education subsequently requested that the educational institutions conduct their courses online, but compared to other nations, the resources and technical capabilities are few [56], which impacts students’ learning. It is understandable that the spread of COVID-19 caused school closures and lockdowns around the world. Adhikari et al. [21] argued that almost all countries experienced changes in education. Since the spread of the COVID-19 pandemic, the universities have transformed teaching and learning, and the lecturers have been conducting online classes. Additionally, the lecturers have delivered their courses via technologies including Zoom, Google Classroom, WhatsApp, and WebEx [12]. Bangladesh started providing COVID-19 vaccines on 27 January 2021, and general immunizations started on 7 February of that same year. University vaccination or Univac was launched to vaccinate university students. After launching vaccines for university students, the government decided to reopen universities. On 27 September, the education minister declared the universities open. The post-COVID-19 education system is similar to the traditional education system as before the pandemic. This study aims to determine how the COVID-19 pandemic has impacted Bangladeshi university students’ ability to learn. The model of transformative learning proposed by Moor is validated by this research. Therefore, the theory of learning begins when learners are confronted with a potentially unpleasant scenario or position. The outbreak caused a paradigm shift in higher education that affected global education systems. The extension of consciousness that occurs as a result of self-adaptation to a changing context is known as transformative learning. According to Moor, a confusing issue makes adjustments that lead to mental aberrations and extreme changes in schooling [7]. 

“Distance education” is a type of education where a significant chunk of the instruction is given by a person who is distant from the learner in both time and space. Although there have been various forms of distance learning since the 1840s, leading academics in the field have attempted to explain distance education theory for decades [57]. A practical framework for distance learning and education is necessary because theories “lead to insights telling us what in distance education is to be anticipated under what circumstances and conditions thus paving the way for corroborated feasible methodological application” [58,59]. To provide an understanding of the possibilities and constraints of facilitating distance learning and teaching using a variety of techniques and technologies is the challenge facing distance education theorists in the twenty-first century [60]. The same is true for online education, just as no single distance educational theory has been developed for instruction in general. Many theories have developed [58,61]. Up until the 1970s, distance education was characterized by a lack of a sound theoretical foundation and largely relied on a trial-and-error methodology [57]. Understandings of the construct and the requirement for a theory inside the context of distance education have been prompted by the need for a strong theoretical foundation. Keegan [61] categorized the different theoretical understandings of distance education and stated that the lack of a self-reliant theory has destabilized distance education into three theories. These are the (i) theory of industrialization, (ii) theory of independence and autonomy, and (iii) theory of interaction and communication. In Keegan’s opinion, general education theory can be used to explain why distance learning is an industrialized form of education instead of a traditional one [61]. Distance education is contradictory, according to Keegan, if education calls for a collective experience in which both teacher and student are linked by a common enthusiasm. Self-directed education and self-regulations are consistent with the idea of independent study [62]. In institutions where structures for the reintegration of the teaching activities are not satisfactorily achieved, distance learners struggle to achieve a good quality of learning [63]. Recently, a wider variety of theoretical ideas has given rise to a stronger understanding of the distance learner. Transactional distance, interaction, learner control, and social presence are four examples of these ideas [58]. According to Moore [64], “transactional distance” refers to the space that separates all educational relationships. The amount of communication between the student and the teacher as well as the degree of structure built into the course’s design both affect this distance.

The concept of interaction is a second theoretical notion that has recently caught the attention of distance educators and has received extensive attention in the theoretical literature. Both traditional and distance education programs depend on the idea of interaction to be effective [63]. Independence and learner control are a third theoretical idea that has received attention in the literature on distance education. According to studies on locus of control, a student who believes that their academic achievement is the results of their own efforts has a sense of internal control that makes them more likely to stay in school [62]. The social environment wherein distance learning occurs is also becoming a significant area of study. The social environment’s impact on motivation, attitudes, teaching, and learning is a topic of theoretical investigation. There is a pervasive belief that technology is societally indifferent and is simple to use in a variety of contexts [58]. Besides these, there are many theories regarding distance learning and education, online learning, and e-learning, such as equivalency theory [58], teaching-learning conversation theory [59], community of inquiry theory [60], transactional distance theory [64], self-regulatory learning theory [65], theory of internalization [66], situated learning theory [67], collaborative learning theory [68], and so on.

These are relevant to this paper because they examine how Bangladeshi students learn and teach during unexpected changes in higher education. The transformative learning concept focuses on an individual’s response to a consistent learning environment. It argues that when students have a positive growth opportunity, they produce meaning, which leads to changes in their attitudes, behaviors, and perception. As students undergo mindset changes, they should be given challenging tasks that require them to think critically and rationally to evaluate their understanding of what is happening [5,12,13]. Moor [64] also stated that transformative learning occurs when learners interact with their surroundings and integrate with the learning process. Learners may face issues with accessing resources in developing countries because the facilities are limited [5]. As a result of this pandemic, students’ academic outcomes in higher education have been impacted. Educators must use effective techniques and adjust learning methods to meet new standards to enhance students’ learning during cognitive dissonance. Therefore, these changes will lead to growth and change in a focused manner. This shift will result in a greater sense of control and awareness.

In this study, we conducted a survey and semi-structured interviews with a certain number of students. To conduct the survey, we developed a 4-point Likert scale and six semi-structured questions to identify the online teaching and learning perceptions of higher education students in Bangladesh. We also discuss our findings along with the findings of some other similar studies conducted globally. Finally, we studied and reviewed the teaching practices following the coronavirus outbreak and examined potential problems related to online learning, and therefore, we also offer recommendations for maintaining the educational system during COVID-19.

## 3. Methodology

**Figure 1:**
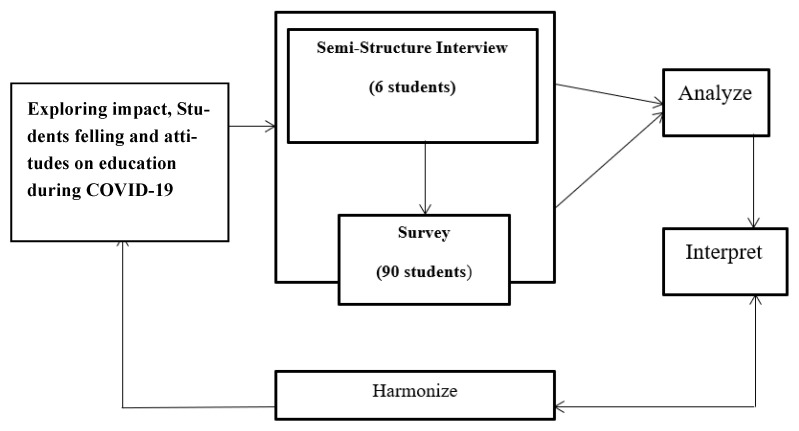
The research data sources and methods.

This section presents the methodology used in our study, including the data collection and analysis processes. The current research is based on both quantitative and qualitative research approaches [15], which were employed to investigate the impact of COVID-19 on the education sector and the perceptions and achievements of students during the pandemic in Bangladesh. According to Chowdhury et al. [15], mixed-method research should place with an equal focus on quantitative and qualitative techniques. An online survey and a semi-structured interview were used to obtain the data. The researcher gathered quantitative data first, followed by qualitative data. Accordingly, we analyzed and summarized the study’s findings. To achieve this, a mixed-methods approach was used, where equal emphasis was given to both types of research. Distance learning during COVID-19 was a necessary period that involved changes in students’ learning activities and educational tools. It is widely acknowledged in research that self-reported data may not accurately reflect a specific problem unless they are combined with reliable information [69]. In this study, respondents were surveyed online and interviewed in a semi-structured manner to collect data. Prior to collecting qualitative data, the researcher gathered quantitative data first, which were then interpreted and used to inform the findings. The study also considered various factors such as age, education, and gender to identify the effects of online learning. To achieve the research objectives, the researchers used three analysis tools, as shown in Figure 1. 

### 3.1. Quantitative Research

#### 3.1.1. Sample Size

The respondents in the quantitative studies were undergraduate and graduate students from a public university in Bangladesh, mainly from Barisal University, Patuakhali Science and Technology University, and Bangabandhu Sheikh Mujibur Rahman Science and Technology University Gopalganj, located in the southern part of Bangladesh. A total of 100 respondents was required for the study from these three universities, with the majority of them coming from the different departments of the Business and Management faculty. Only 90 students accurately completed the survey questionnaire out of the 100 selected respondents. In order to enable scientists to summarize the results of the research for the entire community, a representative sample of the general population was selected for the study. To reduce the sampling error of the results, the researcher selected a broad range of students, as some may not have completed the questionnaire. It is essential to note that the participants’ high response rate means that the missing records have no bearing on how the statistics were interpreted and discovered [70]. Table 1 shows the sample’s demographic information, where females (53.3%) were more numerous than males (46.7%) among survey participants. Most participants’ ages are from 21 to 25 years. Among the participants, 51.6% were undergraduates, and 48.4% were graduates.

#### 3.1.2. Instrument

A survey was conducted by the researcher to develop the study questionnaire [31,56]. A literature review was used to develop the questionnaire items [12,56]. Based on previous literature, an online survey was generated to adopt questionnaire items. An online survey questionnaire was used in data collection. The overview survey comprised of three main segments. The respondents’ demographic background was elicited in the first part. The second segment was divided into three subsections with a total of 19 questions to ask students about their experiences of teaching and learning and the impact of the COVID-19 outbreak on undergraduate studies in higher education. Questionnaires investigating teaching, learning, learner’s performance, students’ objectives, and students’ feelings were analyzed to determine the effects of COVID-19 on the emergence of distant tertiary education in the southern region of Bangladesh (Table 2).

The questions were on a four-point Likert scale ranging from strongly agree (4), agree (3), disagree (2), and strongly disagree (1) [73]. The questionnaire was pilot-tested with 20 students who were not included in the actual study. It was modified after receiving comments from the pilot study. To determine the reliability of the questionnaire, Cronbach’s alpha was used. Overall alpha values for each question were over 0.75, indicating good reliability of the instrument (Table 3).

#### 3.1.3. Data Collection Procedure

To collect the data, we shared our study’s aim with my supervisor and obtained their permission. After that, we distributed the survey link to the participants via messenger and enquired about their willingness to participate in the survey. Before beginning the survey, the respondents had to indicate their agreement by checking a box that confirmed their readiness to take part in the study. Respondents had until 30 July 2022 to complete the questionnaire. The questionnaire took participants around 10–15 min to complete. The application was submitted to the proctorial body of the concerned university for face-to-face interview of the participants. After the approval of the application, the students were interviewed with the permission of the chairman of the designated departments.

#### 3.1.4. Data Analysis 

The data were statistically and thematically examined in this mixed-method research. For each statement, frequencies, percentages, and mean scores are presented through tables. Regarding the quantitative component, we used SPSS to analyze the data The information was obtained from a Google form and changed into an Excel file. After that, the data were imported into SPSS for statistical analysis. There were statistical analyses that were both descriptive and inferential. To determine the frequency, percentage, probability, and mean, a descriptive analysis was conducted. *t*-tests and one-way ANOVA tests were used to determine whether respondents’ responses differed based on their demographic variables, for example, orientation, class, and age. Additionally, the impact of the COVID-19 pandemic on respondents’ perceptions of teaching and learning was also examined through regression analysis [56].

### 3.2. Qualitative Research

The study participants were students from Barisal University, Patuakhali Science and Technology University, and Bangabandhu Sheikh Mujibur Rahman Science and Technology University Gopalganj. For the qualitative study, we selected undergraduate students who lived near the universities. We chose six students to be the test subjects because they had greater tutoring and reading support before and after the COVID-19 pandemic and had more in-depth knowledge than first-time and alternate-time students. Additionally, the students received tutoring and literacy conditioning in the cities and quarters. To gather information from the respondents, we conducted semi-structured interviews. The interview questions were adapted from earlier literature [5,7,8,74] and were also shared with two of our colleagues for feedback and suggestions. The questions were revised and improved based on their input and ideas. The interviews were recorded, and the experimenter transcribed the recordings to assist in analyzing the results of the conversations [75,76].

### 3.3. Ethical Considerations

The students who participated in this study answered the questions independently, and no one was forced to complete the questionnaire. The objective of this study was clearly stated in the questionnaire, and the researcher explained the main objective of the study to the participants before conducting the interview. It is also stated that no monetary award would be given to the study participants. There was an opportunity to ask questions to learn more about the research topic, and it was ensured that any participant would have the opportunity to withdraw if they so desired. A non-objection statement was taken that the participant’s opinion may be used in future research. During the conduct of the present research, the researchers were aware of the interest of the society, institution, individual, or any group and ensured that no harm was done. The study participants’ privacy and confidentiality were fully respected by the researchers, who also took precautions to shield their personal data. Participants gave their permission for the researchers to use and reuse their personal data for research purposes. All the participants and collaborators of this study are thanked for their valuable feedback, time, and effort.

## 4. Data Analysis and Results

To estimate the effect of respondents’ demographic characteristics (age, gender, and education), the study conducted an independent sample *t*-test and one-way ANOVA test. For each variable, the mean was used to analyze the variations in the members’ reactions.

According to the findings of Table 4 (significance value), compared with 0.05, the *p*-value for gender is 0.13. As a result, it can be inferred that the gender of the pupils has no bearing on their replies. According to the findings of the ANOVA test with one factor (significance value), the *p*-value for age is 0.002 and for the education level of the participant is 0.048, both of which are less than 0.05. As a result, the respondents’ education level and their responses were significantly influenced by their age. 

The study obtained information from 90 pupils. Table 5 and Table 6 provide a descriptive investigation of the influence of the COVID-19 pandemic on students’ teaching and learning. 

Table 5 shows that 75.6% of respondents agreed, and 3.3% of respondents strongly agreed with the statement that they had encountered steady access to web-based education during the COVID-19 pandemic in Bangladesh.

However, 5.6% of respondents strongly disagreed, and 12.25% disagreed with the statement that while there was a COVID-19 pandemic in Bangladesh, it was not necessary to have regular access to online instruction. The mean score (2.8) thus indicates that throughout Bangladesh’s COVID-19 epidemic, the pupils had continuous access to online instruction. The above table also shows that 71.1% of the respondents agreed, and 15.6% of respondents strongly agreed with the statement that they were able to contact their teacher through the web-based platform, and the mean score (2.91) indicates that students could easily contact their teacher. However, 74.4% of respondents agreed, and 2.2%) strongly agreed, while 20% disagreed with the statement that they received constructive comments from their lecturers, and the mean score (2.65) indicates that the students had a positive teaching experience during the pandemic.

Table 6 shows that 77.8% of respondents agreed, and 16.7% of respondents strongly agreed with the statement indicating that they had consistent access to web-based education during the COVID-19 pandemic in Bangladesh. However, 0% of respondents strongly disagreed, and 5.6% disagreed with the statement indicating that they did not have experience providing ongoing availability of online instruction during Bangladesh’s COVID-19 epidemic. The mean score (3.11) thus indicates that the students experienced continuous access to online education amid the COVID-19 pandemic in Bangladesh. The table also shows that 64.4% of respondents agreed, 10% strongly agreed, and 22.2% disagreed with the statement, and the mean score (2.81) shows that students had a positive Internet connection during the pandemic.

The indicative data on students’ perceptions of the COVID-19 pandemic’s effects on several aspects of students’ studies in a university in Bangladesh are shown in Table 7, Table 8 and Table 9.

Table 7 shows that 64.4% of students agreed, and 33.3% of students strongly agreed that the COVID-19 pandemic had an impact on how well they learned. However, 2.2% of students disagreed with the statement that the COVID-19 pandemic did not impact their learning performance. The mean score (3.29) thus expresses the negative impact of COVID-19 on students’ learning performance and achievement. It also shows that 73.3% of respondents agreed, 24.4% strongly agreed, and 2.2% disagreed with the statement that the pandemic affected their subject knowledge.

Table 8 shows that 52.2% of students agreed, and 34.4% of students strongly agreed that their future goals were affected by COVID-19. It also shows that 33.3% of respondents agreed, 6.7% strongly agreed, and the mean score (3.65) expresses that COVID-19 delayed their graduation. It also shows that 47.8% of respondents agreed, 50% strongly agreed, 2.2% disagreed, and the mean score (3.48) indicates that the pandemic impacted their educational activities. However, 13.3% of students strongly disagreed that the COVID-19 pandemic did not impact their future goals. The mean score (3.07) thus expresses that COVID-19 negatively impacts students’ future goals.

Table 9 shows that 62.2% of students agreed, and 30% of students strongly agreed that the COVID-19 epidemic affected them psychologically. However, 2.2% of students strongly disagreed, and 5.6% of students disagreed that they were not psychologically affected due to the COVID-19 pandemic. The mean score (3.25) thus indicates that the COVID-19 pandemic harmed students’ feelings. Therefore, the COVID-19 pandemic has adversely affected higher education students’ learning.

Table 10 shows that a positive significant correlation was found between the COVID-19 pandemic and teaching, learning, and student achievement, but a negative significant correlation was found between the COVID-19 pandemic and student goals. There was no relationship found between the COVID-19 pandemic and student feelings. On the other hand, a positive significant correlation was found between teaching and learning, teaching and student achievement, and learning and student goals.

The study utilized regression analysis to research the relationship between students’ teaching and growth opportunities and the effect of the ongoing COVID-19 epidemic on students’ studies in advanced education settings (Table 11).

The test results specify that the *p*-value is 0.001, which is not consistent with the level of significance (0.05). This suggests that learning and teaching are significantly correlated in advanced Bangladeshi education in relation to the impact of the COVID-19 pandemic on students’ learning. 

Qualitative information was gathered through a semi-structured interview with six individuals. The data obtained showed that nearly all participants were dissatisfied with web-based teaching and learning due to several challenges experienced during the COVID-19 pandemic, such as unreliable Internet connectivity, inadequate technology, and financial constraints. A student from the Finance and Banking Department reported that their teachers provided questions for each section of their courses and asked students to research and answer them. However, due to the high cost of Internet packages, she was unable to follow her classes properly. Another student expressed that web-based learning through the ZOOM platform was inadequate, as some students had poor Internet connections, and Internet packages were expensive. He suggested that the Ministry of Higher Education should develop a free web-based platform that can function even with a slow Internet connection. Another participant stated that she faced challenges accessing the web due to a lack of technical resources and financial constraints, which negatively impacted her academic performance (Table 12).

Furthermore, one participant went on to say that his instructors used social media to contact him, but they did not take education and learning seriously. Education and learning should never be stopped, according to the participant. He also expressed that COVID-19 had a negative impact on his mental health, and he was unable to achieve the same high marks as he did in 2019. Additionally, another participant shared that teaching and learning were not fulfilling due to the COVID-19 pandemic affecting her concentration and the institutions closing for a prolonged period of time. During the pandemic, the students were also concerned about the fact that they would experience added pressure due to shorter terms, constant classes, sessional tests, and having to prepare for the term finals with short notice. Due to a lack of education and learning, one participant reviewed his notes and studied at home. He also mentioned that it was common for students to not study effectively when teachers did not push them, which ultimately affected their learning outcomes. Finally, almost all the participants were dissatisfied, as their learning outcomes were negatively impacted by problems with the experience of online teaching and learning as well as issues with the Internet and technological infrastructure.

## 5. Discussion

The noxious effects of the COVID-19 pandemic have been felt all over the world and have spread rapidly. The COVID-19 pandemic is affecting the education sector just as it is affecting other sectors. Students’ university education is not exempt, and learning is no exception. The COVID-19 pandemic has challenged the education community. From pre-primary to higher education, 1.725 billion children and young people in 200 countries have been impacted by the pandemic as of July 2020, accounting for 98.6% of all learners worldwide [18,19]. Governments of many developing nations including Bangladesh have been unable to cope with the transition due to limited resources and inadequate infrastructure [9,77]. The objective of this research was to examine how the COVID-19 pandemic affected student learning in Bangladeshi higher education institutions. It also aimed to determine if student demographic characteristics such as orientation, social position, and age had any bearing on their responses. The pandemic has forced a significant shift away from learning and teaching in traditional settings with physical interactions. Online learning is a newer and uncommon approach in developing countries such as Bangladesh [9,78]. Study findings indicate that some students did not receive effective and consistent online learning and instruction during the COVID-19 epidemic due to a lack of facilities and resources. Students’ engagement in higher education has been hindered by these limitations [18,19].

As a result of a shortage of resources, students faced many obstacles in teaching and learning activities during COVID-19, which hindered their learning [23,79]. The level of academic performance of students is likely to drop for both year-end and internal examinations due to reduced contact hours for learners and a lack of consultation with teachers when facing difficulties in learning/understanding [25,80].

Our findings also show that the prolonged closure of the university adversely impacts the scholarly life as well as the personal lives of the students. A handful of university students participated in confidential tuition to help themselves and, to some extent, their families. This prolonged lockdown seriously impacted their income from beneficial classes that, in some cases, caused mental pressure. Previous studies also suggest that financial solvency is a motivating factor to survive during crises [1,80,81,82]. The results of the quantitative survey show that most students have a favorable impression of their teachers’ teaching, and they stated their teachers had been cooperative during online classes (Table 5). Most students’ learning perceptions are also positive, as we found that online classes help them learn about various technological resources (Table 4).

Despite these positive perceptions, during the COVID-19 pandemic, a significant number of students was dissatisfied with online teaching and learning, which indicates the lowest percentage (Table 5 and Table 6). They said that the majority of learners had problems using the Internet and other technology. For instance, Participant 2 reported that his local 3G network was down, so he was unable to follow his lectures using only 2G Internet [1,83,84,85,86]. The participants also mentioned that they were having trouble paying for broadband bundles because most of them were prohibitively expensive. Moreover, the mobile network’s Internet was unreliable, and they could not participate in competent online learning and recovery during the COVID-19 epidemic.

Subsequently, they recommended that the Department of Higher Education of Bangladesh reassess its procedures and create and distribute a suitable web-based platform tailored to Bangladesh. Participant 2 suggested, as an illustration, a management system for learning that is free for students and is compatible with sluggish Internet connections. The outcome statement upholds a review that demonstrated Internet education and learning to be inadmissible because of an absence of sufficient facilities. Additionally, the COVID-19 epidemic has hindered students’ academic progress in higher education.

The data were analyzed, and conclusions show that nearly 90% of respondents said the viral epidemic and their learning were significantly impacted by confinement (Table 7, Table 8 and Table 9). The study’s participants found evidence of the COVID-19 pandemic’s effects on various aspects of students’ learning, including class projects, assignment loads, learning quality, motivation for learning, educational activities, goals, subject knowledge, learning performance, educational opportunities, and the length of their studies. This outcome corroborates the findings of previous research [5,87]. The effects of anxiety on many pupils’ learning and academic performance have been detrimental. According to this evaluation, the provision of counseling and mentoring services by educational institutions is essential for students to succeed in university education. The review’s conclusions also showed that respondents’ orientation had a major influence on their reactions (Table 4).

COVID-19 has had a significant impact on Bangladeshi university education and students’ learning, including their opinions of online education (Table 10 and Table 11). Some students face a lack of access to resources during online learning due to poor network connectivity and power cuts, leading to anxiety and frustration [88]. These challenges can result in poor academic performance and strained relationships. Therefore, educational institutions should strive to provide students with adequate materials for teaching and learning over the Internet [4,89,90,91]. The COVID-19 pandemic and teaching, learning, and student achievement have been found to have a positive significant correlation, but the COVID-19 pandemic and student goals have been found to have a negative significant correlation according to the results. There is no connection between student emotions and the COVID-19 pandemic. The morale and tenacity of Bangladeshi students was unwavering during the COVID pandemic. COVID-19 did not divert most of the students from their life goals and education goals. Amidst the disruptions of COVID-19, students have continued to study to pursue their goals [87,92,93].

Qualitative findings from this study show that COVID-19 has adversely affected students’ learning in higher education (Table 12). The pandemic has presented students with unique challenges, but it has also allowed them to quickly learn new information through online education. Nevertheless, many students reported that the pandemic has had a detrimental effect on their academic achievement, causing them to miss graduation, lose their jobs, and experience financial difficulties. These results align with those of other researchers who found that COVID-19 has impacted students’ learning [20,31].

The study also highlights students’ concerns about delayed graduation, which may affect their future professional careers. Graduating on time would bring societal and financial benefits to the students and their families, leading to a better quality of life and living standards [27,94]. However, the study has some limitations; it only focuses on the public university students’ learning system and their perceptions and experiences of online classes. The study covers only three departments of a public university in Bangladesh and does not explain other factors, such as students’ psychological conditions during the pandemic and how they can handle the situation and make up for their academic losses. Nevertheless, the study indirectly addresses these problems that need to be solved.

## 6. Conclusions

The COVID-19 pandemic has brought global change since the World Health Organization declared it a global pandemic in March 2020. This has resulted in a significant shift in teaching and learning for college students globally, and Bangladeshi students have not been an exception. Due to low resources, Bangladeshi children’s education has been negatively impacted more than any other nation. Most students lack consistent teaching and learning activities, have weak Internet connections, have limited technological facilities, and have high-cost Internet, making online learning difficult. Students often drop semesters as they worry about their futures and struggle to attend online exams effectively. Zoom, Google Meet, and Google Classroom have been used to teach optional, higher auxiliary, and college students online. This study’s participants understand COVID-19’s risks and treatments, but the delay in evaluating them has caused them to worry about their futures as scholars and experts. College students may be psychologically troubled by the impending meeting restrictions and a doubtful career.

Since most students and teachers had never taken a web-based class before, it was difficult to get everyone involved and give good class introductions. Lack of mechanical information, correspondence, and long lectures were the biggest challenges for instructors. Bangladesh, a developing nation, has suffered more from the COVID-19 pandemic than other countries that had a better response. The UGC of Bangladesh awarded poor students repayable delicate credits for online class computer purchases. Long-term lockdowns also affected school dropouts, child labor, and early marriage. Contrary to expectations, last year’s university undergraduates failed to graduate and missed out on many job opportunities. The COVID-19 pandemic has forced all schools to teach online. States should scale network foundations and web availability throughout metropolitan and provincial regions. The nations should scale instructional innovation, lay down zero-rating instructional resources on the web, prepare computerized teaching and learning resources, use free Internet learning resources, use mobile learning, use radio and TV education, and expand ICT frameworks. Scientists, educational program architects, school authorities, and instructional foundations should work to reform education during lockdowns. Schools and universities should plan how to teach and learn after COVID-19, and the school system should be reorganized. After COVID-19, schools and universities should seek ways to recover lost segments, get children back to school, and scale online learning. Finally, the COVID-19 pandemic is affecting schools in non-industrial nations. Thus, underdeveloped nations should increase online education services. Radio and TV broadcasting, data, and communication technology can help developing nations enhance education. It also requires computerized instructor training.

According to the report, some students think their online teachers are cooperative, knowledgeable, and have good technology. All students worry about their professions, grades, and semester drops. The COVID-19 pandemic has negatively impacted students’ learning; many feel like they did not learn for the year. Age, gender, and education did not differ in this study either. The study found that the COVID-19 pandemic has had beneficial and harmful effects on higher education students’ teaching and learning. Web-based classes are not practical for state-funded college students. Most Bangladeshi college students from low-income households do not have the means to spend money on Internet providers to attend online classes, especially during the epidemic. Lower-middle-class households struggle financially and eventually cannot afford essentials. The researchers also expected different outcomes for private college students. They noticed that private college students are primarily from affluent households, while state-funded college students can only afford lower tuition. The policymakers of the educational sector and the UGC (University Grants Commission) should take into account all facets of the higher education system and adapt and adjust policies as necessary. For instance, during the COVID-19 pandemic, students experienced significant changes that frequently negatively impacted their learning at some other southern-region universities.

### 6.1. Implications

COVID-19 has become a pandemic in recent days. Undoubtedly, the overall scenario of the Bangladesh education system has changed because of COVID-19. The goal of this research was to learn out about the higher education learning system during COVID-19. Study findings have found that although most of the students had positive higher education experiences during the epidemic of COVID-19, some students had negative experiences. However, the pandemic has truly had a negative impact on the learning system. Though online classes are being held, students face many problems such as poor exam results, dropping out of semesters, low GPA or CGPA, and a general lack of interest in online classes. They prefer traditional face-to-face classes. However, it can be said that in the worst situations in a country, continuing education programs through online platforms is the best solution. Therefore, the findings of the study help education policymakers understand the overall scenario of the learning system during COVID-19. The findings suggest that teachers should be trained in the use of technology so that they can cope with worse situations. Besides this, the country needs to improve its broadband Internet connections so that students in rural areas can have strong network connections.

### 6.2. Limitations and Future Research Direction

This study has certain limitations. The major limitations of the study include the size and nature of the samples. The estimated sample size for the study is too small to generate conclusive findings. The data were collected only from one department of a public university in Bangladesh, and thus, the study’s findings may not be generalizable to other institutions. Additionally, this study did not explore the teachers’ perceptions during the COVID-19 learning system, which could have helped identify the actual gap between the students’ and teachers’ perceptions. Furthermore, this study did not offer a comprehensive solution to the problem. Further research is needed to reduce the study’s limitations, as this study is limited by insufficient data and a small sample size. Future researchers should consider the limitations of this study and conduct further research on this issue.

## Figures and Tables

**Table 1:** Sample size distribution of participants.

Demographic Variable		Frequency	Percentage
Gender	Male	43	47%
	Female	47	52%
	Total	90	100%
Age	21 to 25	65	71.1%
	26 to 30	25	28.9%
	Total	90	100%
Level of education	Undergraduate	45	50%
	Graduate	45	50%
		90	100%

Source: Authors’ analysis.

**Table 2:** Overview of the measurement items of the questionnaire.

Teaching	**Statement**	**Items**	**References**
	Despite the COVID 19 outbreak, my teachers continued to teach as usual.	T1	[5,12,13,31,56]
	I had contact with my teachers through a web-based platform.	T2	
	I receive constructive feedback from my lectures.	T3	
	I receive support from my lecturers.	T4	
	Online classes were very effective for me.	T5	
Learning	I approached web-based classes during the COVID-19 pandemic.	L1	[12,13,31,71]
	I was connected to the Internet during the COVID-19 pandemic.	L2	
	I had a tech installation during the COVID-19 pandemic.	L3	
	I am using various resources during the COVID-19 pandemic.	L4	
	I was better acquainted with the use of technology.	L4	
Learners’ performance	The COVID-19 pandemic impacted my learning performance.	LP1	[1,5,12,13]
	My subject expertise was influenced by the COVID-19 pandemic.	LP2	
	The COVID-19 pandemic affected the nature of my learning.	LP3	
Students’ objectives	The COVID-19 outbreak has altered my plans for the future.	SO1	[12,13,31,56]
	The COVID-19 pandemic delayed my graduation.	SO2	
	My educational activities were affected by the COVID-19 pandemic	SO3	
Student’s feelings	Because of COVID-19, I believe I didn’t read up for quite a long time.	SF1	[1,56,72]
	The COVID-19 pandemic impacted me psychologically.	SF2	
	I believe I lost instructive open doors during the COVID-19.	SF3	

**Table 3:** Reliability value of questionnaire items.

Category	Number of Items	Cronbach’s Alpha
Teaching	5	0.881
Learning	5	0.862
Students’ Achievements	3	0.791
Students’ Goals	3	0.775
Students’ Feelings	3	0.758
Overall	19	0.894

**Table 4:** Respondents’ demographic characteristics.

Variable		N	Mean	Standard Deviation	*p*-Value
Gender	Male	43	2.55	0.296	0.13
	Female	47	2.53	0.215	
Age	21–25	65	2.535	0.304	0.002
	26–30	25	2.606	0.353	
Education Level	Undergraduate	45	2.597	0.212	0.048
	Graduate	45	2.566	0.259	

**Table 5:** Teaching.

Statement	SD	D	A	SA	Mean
T1	5.6%	12.2%	75.6%	3.3%	2.82
T2	0	6.7%	71.1%	15.6%	2.91
T3	0	20%	74.4%	2.2%	2.65
T4	0	13.3%	78.9%	4.6%	3.11
T5	3.3%	38.9%	41.1%	13.3%	2.81

Note: SD, strongly disagreed; D, disagreed; A, agreed; SA, strongly agreed.

**Table 6:** Learning.

Statement	SD	D	A	SA	Mean
L1	0	5.6%	77.8%	16.7%	3.11
L2	3.3%	22.2%	64.4%	10%	2.81
L3	0	16.7%	77.8%	5.6%	2.89
L4	0	18.9%	67.8%	11.1%	2.92
L5	3.3%	38.9%	41.1%	13.3%	2.653

Note: SD, strongly disagreed; D, disagreed; A, agreed; SA, strongly agreed.

**Table 7:** Effect of the COVID-19 epidemic on learners’ performance.

Statement	SD	D	A	SA	Mean
LP1	0%	2.2%	64.4%	33.3%	3.29
LP2	0%	2.2%	73.3%	24.4	3.20
LP3	0%	3.3%	65.6%	31.1%	3.24

Note: SD, strongly disagreed; D, disagreed; A, agreed; SA, strongly agreed.

**Table 8:** Effect of the COVID-19 epidemic on students’ objectives.

Statement	SD	D	A	SA	Mean
SO1	13.3%	0%	52.2%	34.4%	3.07
SO2	0%	0%	33.3%	6.7%	3.65
SO3	0%	2.2%	47.8%	50.%	3.48

Note: SD, strongly disagreed; D, disagreed; A, agreed; SA, strongly agreed.

**Table 9:** Impact of COVID-19 pandemic on student’s feelings.

Statement	SD	D	A	SA	Mean
SF1	0%	10.%	54.4%	35.6%	3.48
SF2	2.2%	5.6%	62.2%	30%	3.25
SF3	0%	16.7%	41.1%	40.1%	3.20

Note: SD, strongly disagreed; D, disagreed; A, agreed; SA, strongly agreed.

**Table 10:** Correlation matrix among independent variable (COVID-19) and dependent variables.

Variables.	1	2	3	4	5	6
1. COVID-19 Pandemic	1					
2. Teaching	0.438 **	1				
3. Learning	0.291 **	0.0361 *	1			
4. Students’ Achievements	0.126 *	0.118 **	0.116	1		
5. Students’ Goals	−0.328 **	0.127	0.314 *	0.138	1	
6. Students’ Feelings	0.221	−0.241	0.0.112	0.123	−0.231	1

* Correlation is significant at the 0.05 level (2-tailed); ** Correlation is significant at the 0.01 level (2-tailed).

**Table 11:** Inferential Analysis (Regression).

Model	Sum of Square	df	Mean	F	Sig
Regression	1.883	2	0.941	1.605	0.005
Residual	51.017	87	0.586		
Total	52.900	89			

**Table 12:** Overview of qualitative data source based on interview regarding COVID-19.

“Themes”	“Participant 1”	“Participant 2”	“Participant 3”	“Participant 4”	“Participant 5”	“Participant 6”
Students’ experiences of teaching during COVID-19	“Our professors established chat groups in which they shared stuff.”	“I lived in a part of town and couldn’t attend my classes most of the time.”	“Since numerous Understudies didn’t have a cell phone or a PC, online talks were incapable for them.”	“Our professors used to contact us via ZOOM and would send us recordings of their lectures.”	“I wasn’t happy with the teaching because we hadn’t had any lectures in months.”	“Lecturers used to use social media to communicate and share some lesson resources.”
Students’ experiences of learning during COVID-19	“Since most of the understudies didn’t approach the Internet, the web-based addresses ceased after a couple of meetings.”	“Web-based learning was incapable for me because of an absence of Internet access and innovative issues.”	“I used to make phone calls to my classmates and ask them for the tasks that the lecturers had assigned.”	“Because our instructors were not serious about their teaching, the learning experience was not favorable.”	“Coronavirus hurt my learning style and fixation when it came to learning new things. I teamed up with a colleague on a class project.”	“I used to rehash my sections and notes; however, my learning result was poor because the educator put no squeeze on us to concentrate sufficiently on”
Challenge	“Sometimes I didn’t have power, and most of the time I had Internet issues.”	“I had difficulty with the Internet and electricity.”	“I had financial difficulties and was unable to get a smartphone in 2020, and as a result of which I was unable to study efficiently.”	“We had extremely slow Internet and were unable to download the shared information.”	“Our university was closed, and we were unable to attend classes. Have months of class.”	“Have no class this month.”
Solutions	“The Ministry of Higher Education ought to foster a fantastic learning programming”	“The Ministry ought to present a product which will be wide open and work with slow Internet because In the locale, we have 2G Internet as it were.”	“The Ministry ought to lay out a strategy for monetarily burdened understudies.”	“Teaching and learning should never be interrupted, and the Ministry can design a practical and effective learning application.”	“In emergencies, such as the COVID-19, instructors and lecturers should encourage students and check their learning progress.”	“The Ministry of Higher Education ought to plan and execute a web-based stage that is available to all understudies and for nothing.”
The Impact of COVID-19 on students’ learning.	“Coronavirus made both positive and adverse consequences, albeit the last option unfavorably affected my learning.”	“As a result of the horrendous Internet, I had the most exceedingly terrible opportunity for growth in my life, I was unable to try and peruse messages on courier and What’s app.”	“I was under a lot of stress and felt like I hadn’t studied in years.”	“Due to my less than stellar scores because of COVID-19, my graduation was delayed, and I was worried about my well-being and future.”	“The good consequence is that we had our first encounter with online learning, which introduced us to online learning.”	“Coronavirus delayed my graduation, I lost my employment as an educator in an instructive focus, and I was focused on during COVID-19.”

## Data Availability

Not applicable.
